# Global analysis of the abundance of AU-rich mRNAs in response to glucocorticoid treatment

**DOI:** 10.1038/s41598-024-51301-6

**Published:** 2024-01-09

**Authors:** Zeyad Muazzen, Walid Moghrabi, Tala Bakheet, Linah Mahmoud, Maher Al-Saif, Khalid S. A. Khabar, Edward G. Hitti

**Affiliations:** Molecular BioMedicine Department, Research and Innovation, King Faisal Specialist Hospital and Research Centre, 11211 Riyadh, Saudi Arabia

**Keywords:** Transcriptomics, Systems analysis

## Abstract

Glucocorticoids (GC) like dexamethasone (Dex) are potent anti-inflammatory agents with diverse cellular functions including the potentiation of the activity of AU-rich elements (AREs). AREs are cis-acting instability sequence elements located in the 3′UTRs of many inflammatory mediator mRNAs. Here, available RNA-seq data were used to investigate the effect of GCs on the ARE-mRNA-transcriptome. At a global scale, ARE-mRNAs had a tendency to be downregulated after GC-treatment of the A549 lung cancer cell-line, but with notable cases of upregulation. mRNA stability experiments indicated that not only the downregulated, but also the upregulated ARE-mRNAs are destabilized by Dex-treatment. Several of the most upregulated ARE-mRNAs code for anti-inflammatory mediators including the established GC targets *DUSP1* and *ZFP36*; both code for proteins that target ARE-containing mRNAs for destruction. GCs are widely used in the treatment of COVID-19 patients; we show that ARE-mRNAs are more likely to regulate in opposite directions between Dex-treatment and SARS-CoV-2 infections compared to non-ARE mRNAs. The effect of GC treatment on ARE-mRNA abundance was also investigated in blood monocytes of COVID-19 patients. The results were heterogeneous; however, in agreement with in vitro observations, *ZFP36* and *DUSP1* were often amongst the most differentially expressed mRNAs. The results of this study propose a universal destabilization of ARE-mRNAs by GCs, but a diverse overall outcome in vitro likely due to induced transcription or due to the heterogeneity of COVID-19 patient’s responses in vivo.

## Introduction

Inflammation is a highly regulated intracellular and extracellular process. At the cellular level, a complex network of signaling pathways regulate the transcription and post-transcription of inflammatory mediators^1^. At the post-transcriptional level, the function of AU-rich elements (AREs) located in many inflammatory mRNAs is of particular importance. AREs are composed of a single or overlapping repeat(s) of the AUUUA pentamer located in the 3′UTR of up to 22.4% of human mRNAs^2^. AREs repress translation and confer instability of cis-mRNAs. They can also respond to inflammatory stimuli especially the p38/MK2/ZFP36 MAP Kinase phosphorylation pathway that is triggered during inflammation^3^. ZFP36 also called Tristetraprolin (TTP) is an ARE-binding protein that inhibits mRNA translation and induces its decay. At the onset of inflammation, ZFP36 is strongly induced but also immediately inhibited through phosphorylation by p38/MK2. This allows the stabilization and translation of inflammatory cytokines mRNAs early on during inflammation, but also, ensures their decay later on by de-phosphorylated and re-activated ZFP36^4–7^. This “interrupted” negative feedback loop ensures an efficient but transient expression of inflammatory mediators to initiate inflammation. The phosphatase DUSP1 that is also induced during inflammation is a component of this negative feedback loop since it de-phosphorylates and deactivates p38 leading to the de-phosphorylation and re-activation of ZFP36^8–11^. Ultimately, the ARE-dependent post-transcriptional process allows an efficient but transient expression of master inflammatory cytokines like TNF and IL1^7,12^.

Negative feedback loops in inflammation are not restricted to intracellular mechanisms. At the organism level, TNF and IL1 also induce the production and release of glucocorticoids (GCs) into the bloodstream by the hypothalamic–pituitary–adrenal axis to end inflammation; their anti-inflammatory effects are complex and affect several types of cells and organs^13^. Synthetic GCs such as Dexamethasone (Dex) are widely used clinically to treat allergies, autoimmunity, chronic inflammation and COVID-19^14,15^.

Importantly, GCs trigger cellular events that target AU-rich elements, for instance, they induce ZFP36. However, unlike inflammatory stimuli, GC-induced ZFP36 protein should remain un-phosphorylated and therefore active. GCs do not co-activate p38 signaling, instead they induce the phosphatase DUSP1, which reduces p38 MAPK activity. Active ZFP36 that is induced by GCs binds AREs of inflammatory mRNAs leading to reduced translation, enhanced decay, and subsequently reduced inflammation without interruption^9,16–21^. In fact, it has been proposed that post-transcriptional control plays a major effect in GC response, since the Knock-Out of *Zfp36* in mice affects up to 85% of genes whose expression are modulated by GCs^22^.

Here, the analysis of available transcriptomic RNAseq data allowed a global view and improved understanding of the effect of GC treatment on ARE-mRNAs abundance in human cells. Comparative analysis between transcriptomic responses to GCs and to SARS-CoV-2 infections suggest that ARE-dependent post-transcriptional response is a likely factor in COVID-19 treatment.

## Results

### A global analysis of ARE-mRNA response to Dex-treatment

**Figure 1:**
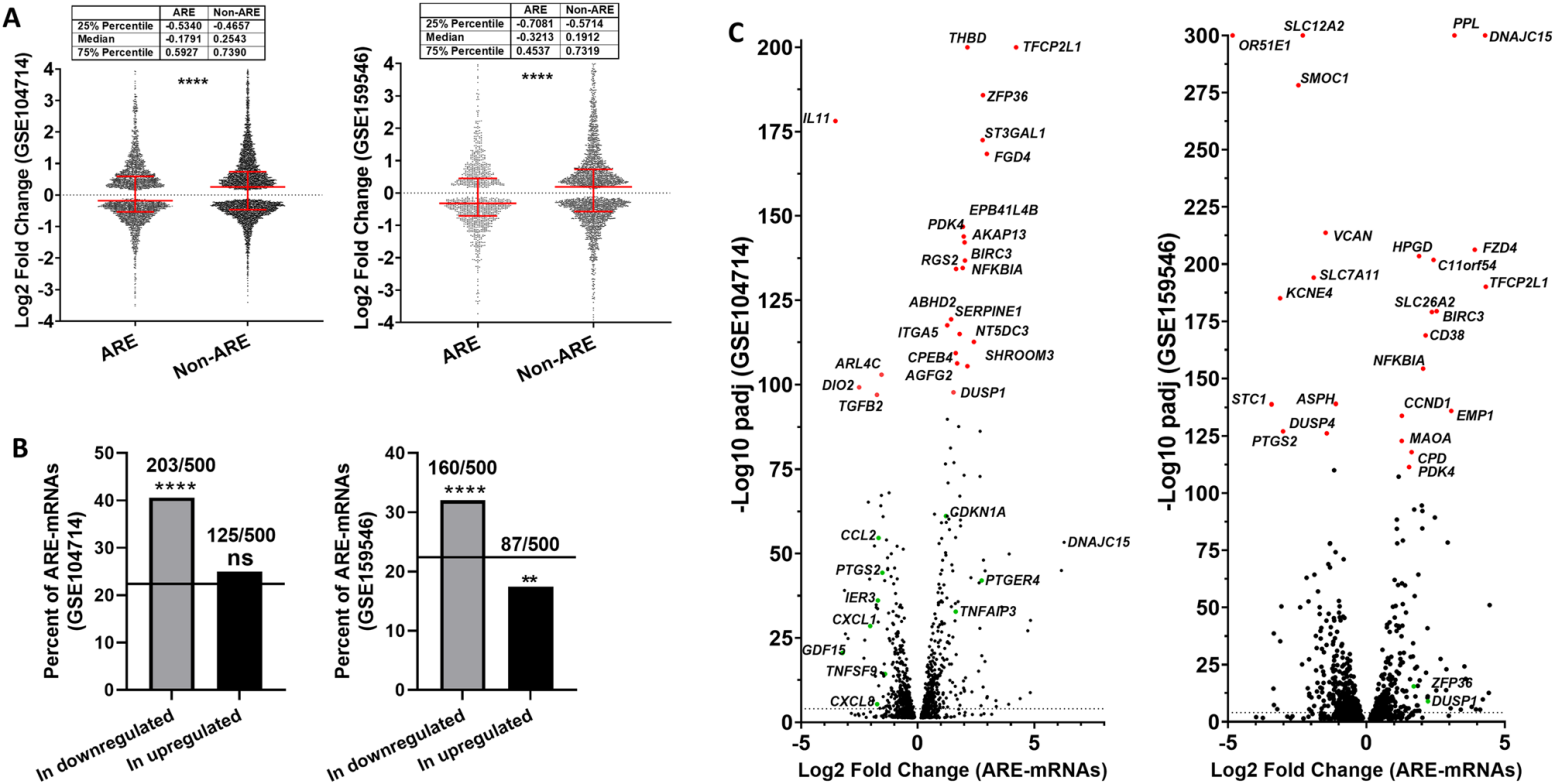
Global analysis of AU-rich mRNA response to GCs in A549 cells. Available RNA-seq data from GSE104714 or GSE159546 were analyzed in DESeq2 to select significantly differentially expressed mRNAs after Dex or hydrocortisone treatment of A549 cells (padj < 0.05). The transcripts were split between ARE-containing and non-ARE. (**A**) Dex-dependent Log2 Fold Change dot plot is displayed with median and interquartile range of ARE and non-ARE mRNAs, Man-Whitney test **** P < 0.0001. (**B)** Displayed is the percent of ARE-mRNAs within the 500 most upregulated transcripts and the 500 most downregulated transcripts by Dex. The line at 22.4% represents the percent of ARE-mRNAs in the whole transcriptome. The significance of the difference in population proportions was tested by two sided fisher’s exact test P < 0.0001 ****, P > 0.05 ns. (**C**) Volcano plots of ARE-mRNAs that are significantly differentially expressed in response to GC-treatment. In red are the 25 most significantly differentially expressed ARE-mRNAs. Other significantly differentially expressed mRNAs that are previously reported to be modulated by GC treatment or targeted by ZFP36 are labelled in green.

Published RNAseq data were used to investigate the global response of ARE-mRNAs to Dex-treatment in the A549 lung cancer cell-line. MacDowell et al. treated A549 cells with Dex for several time points in replicates along with time specific vehicle controls (GSE104714)^23^. The DESeq2 software was used to identify transcripts that were differentially expressed in A549 cells treated for 3, 5, 7, 9, and 11 h by combining all time points and replicates in a single paired analysis. This allowed the identification of transcripts that were differentially expressed across the entire time course. DEGs with Padj < 0.05 (Benjamini and Hochberg adjustment for multiple testing), from DESeq2 results, were considered differentially expressed with statistical significance. In an independent similar investigation, A459 cells were treated with another GC, hydrocortisone, for 8 h in duplicate, DESeq2 was again used to identify differentially expressed genes (GSE159546)^24^. The differentially expressed transcripts from both datasets were crossed with the ARED-plus database to select the ARE-mRNAs (Supp. Tables S1 and S2). The Log2 Fold Changes (Log2FC) of ARE and non-ARE-mRNAs were plotted (Fig. 1A). ARE-mRNAs had a tendency to be downregulated at a global scale compared to non-ARE mRNAs (Medians from GSE104714: ARE Log2FC − 0.17 (25th Percentile − 0.53, 75th Percentile 0.6) versus non-ARE 0.25 (25th Percentile − 0.47, 75th Percentile 0.74), and Medians from GSE159546: ARE − 0.3213 (25th Percentile − 0.7081, 75th percentile 0.45) versus non-ARE 0.19 (25th Percentile − 0.57, 75th percentile 0.72). Next, we determined the fraction of ARE-containing transcripts within the 500 most upregulated and 500 most downregulated transcripts. It turned out that from GSE104714 dataset 25% (125/500 mRNAs) of the upregulated group are ARE-mRNAs while 40.6% (203/500 mRNAs) of the downregulated group are ARE-mRNAs. The 40.6% is a highly significant enrichment of ARE-mRNAs compared to the 22.4% in the whole transcriptome (fisher’s exact test p.val < 0.0001), again indicating a preferential downregulation of ARE-mRNAs by Dex-treatment. Similar observations were made with the GSE159546 (Fig. 1B).

Unexpectedly, a volcano plot shows that 21 of the 25 most significantly differentially expressed ARE-mRNAs after Dex-treatment are upregulated in the GSE104714 (Fig. 1C, left panel). The combined above observations indicate a general global tendency of ARE-mRNAs to be downregulated in response to the glucocorticoid treatment, but with few cases of extreme upregulation. Several of the observed most upregulated ARE-mRNAs are well-established glucocorticoid targets like *ZFP36, DUSP1* and *NFKBIA* that have anti-inflammatory functions and can be considered a validation of the RNA-seq data^19,22,23,25^. Several of significantly downregulated ARE-mRNAs are also known or expected pro-inflammatory GC targets like *IL11, CXCL1, CXCL8, PTGS2, TNFSF15* and *CCL2* (Fig. 1C, left panel)^26–28^. Also, several of the ARE-mRNAs that were modulated by Dex-treatment in A549 cells were previously identified as top 20 ZFP36/TTP targets, like *DUSP1, GDF15, TNFAIP3, NFKBIA, IER3, TNFSF9, CDKN1A* and *CCL2* (Fig. 1C)^11,29^. A volcano plot from GSE159546 dataset contains genes that were similarly differential regulated like *DNAJC15, NFKBIA, BIRC3, PTGS2, TFCP2L1, ZFP36* and *DUSP1* (Fig. 1C, right panel). The two independent datasets had comparable results. Further investigations were based on dataset GSE104714 due to the higher number of replicates over a time course.

### Both the upregulated and downregulated mRNAs are destabilized by Dex

**Figure 2:**
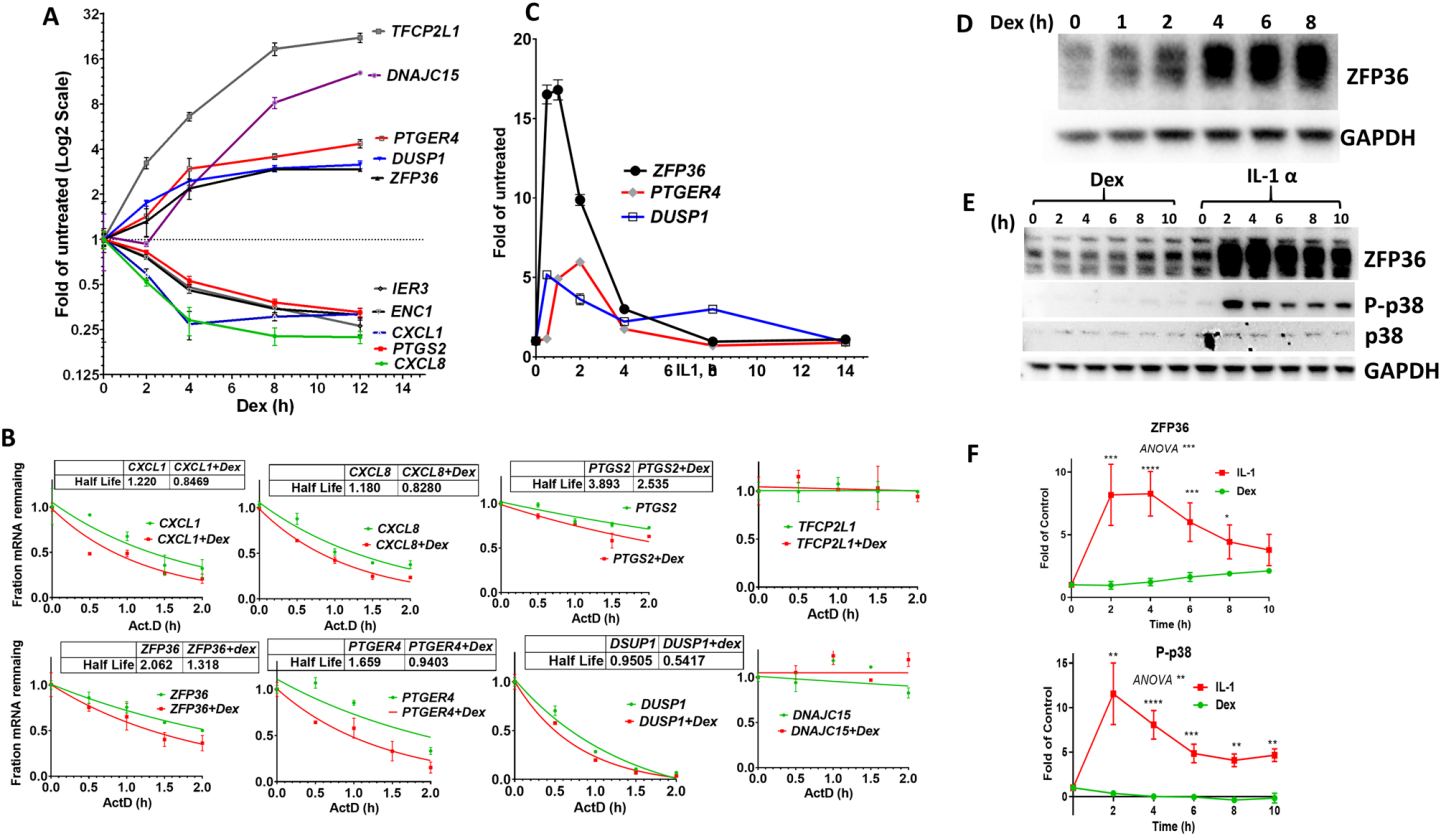
Steady-state levels and stability of ARE containing mRNAs after Dexamethasone treatment. (**A**) A549 cells were treated with Dexamethasone (Dex) for the indicated time points. The relative levels of ten mRNAs were evaluated by qRT-PCR. The data is shown in fold of untreated 0 h control in Log2 scale. (**B**) Actinomycin-D chase experiments for the assessment of mRNA stability 2 h after Dex-treatment. (**C**) A549 cells were treated with 3ng/ml IL1 for indicated time points, mRNAs of *ZFP36*, *PTGER4* and *DUSP1* were measured by real time PCR. (**D**) A549 cells were treated with 100nM of Dex for the indicated time points and western blot analysis was performed to assess ZFP36 and GAPDH levels. (**E**) A549 cells were treated with either 100 nM of Dex or 3 ng/ml IL1 for the indicated time points and western blot analysis was performed to assess ZFP36, phosphorylated p38 (P-p38), p38 and GAPDH levels. Uncropped western blots available supplemental file F1 (**D**) The western blot experiments were repeated three times and ZFP36 and P-p38 bands were quantified by Image Lab (BioRad) and normalized to GAPDH. Mean with SEM are displayed. Two way ANOVA with Dunnett’s repeated measures test (For each time point compared to DMSO control) with* p < 0.05, ** p < 0.01, *** p < 0.001, **** P < 0.0001.

Dex-treatment leads to the destabilization of ARE-containing mRNAs of *TNF* and *CXCL8*, and *PTGS2* in cancer cells^16–18,21^. This is in agreement with the downregulating effect observed here; *CXCL8* and *PTGS2* were amongst the significantly downregulated mRNAs (Fig. 1C, left panel). The RNAseq observations were reconfirmed by a qRT-PCR time-course with candidate ARE-mRNAs that were down-regulated by Dex like *CXCL8, PTGS2, CXCL1, ENC1* and *IER3* (Fig. 2A). Some upregulated ARE-mRNAs were also selected like *ZFP36, DUSP1, PTGER4, DNAJC15* and *TFCP2L1* and their upregulation was also confirmed by qRT-PCR (Fig. 2A). *ZFP36, DUSP1, PTGER4* were modestly upregulated for two and up to fourfold. While the upregulation of *DNAJC15* and *TFCP2L1* was stronger up to 12- and 22-fold respectively (Fig. 2A). Next, the stability of the selected mRNAs was investigated by Actinomycin-D chase experiments. In agreement with previous reports, the stability of *CXCL8, CXCL1*, and *PTGS2* mRNAs was reduced by Dex-treatment. However, the moderately upregulated transcripts *DUSP1, ZPP36* and *PTGER4* were also destabilized by Dex-treatment. The highly upregulated *DNAJC15* and *TFCP2L1* mRNAs had very stable transcripts, suggesting that their predicted AREs are not functional and subsequently not affected by Dex-treatment (Fig. 2B). *DUSP1* and *ZFP36* can be induced by inflammatory stimuli like IL1^30,31^. For comparison, A549 cells were treated with IL1 and a time course of *ZFP36, DUSP1* and *PTGER4* mRNA levels was assessed by qRT-PCR. Their inductions by IL1 were stronger but transient (Fig. 2A,C). In agreement, the induction of ZFP36 protein by IL1 was also transient but much stronger than Dex induction, as assessed by western blot analysis (Fig. 2D–F). As expected, IL1 but not Dex led to the phosphorylation of p38MAPK, implying that Dex-induced ZFP36 is not phosphorylated and active (Fig. 2E,F). The observations suggests that the very low level of Dex-induced ZFP36 is sufficient to trigger increased mRNA decay, in agreement with a previous observation^5^.

### ARE-mRNAs that are downregulated by Dex tend to be upregulated by SARS-CoV-2 infection in A549 cells

**Figure 3:**
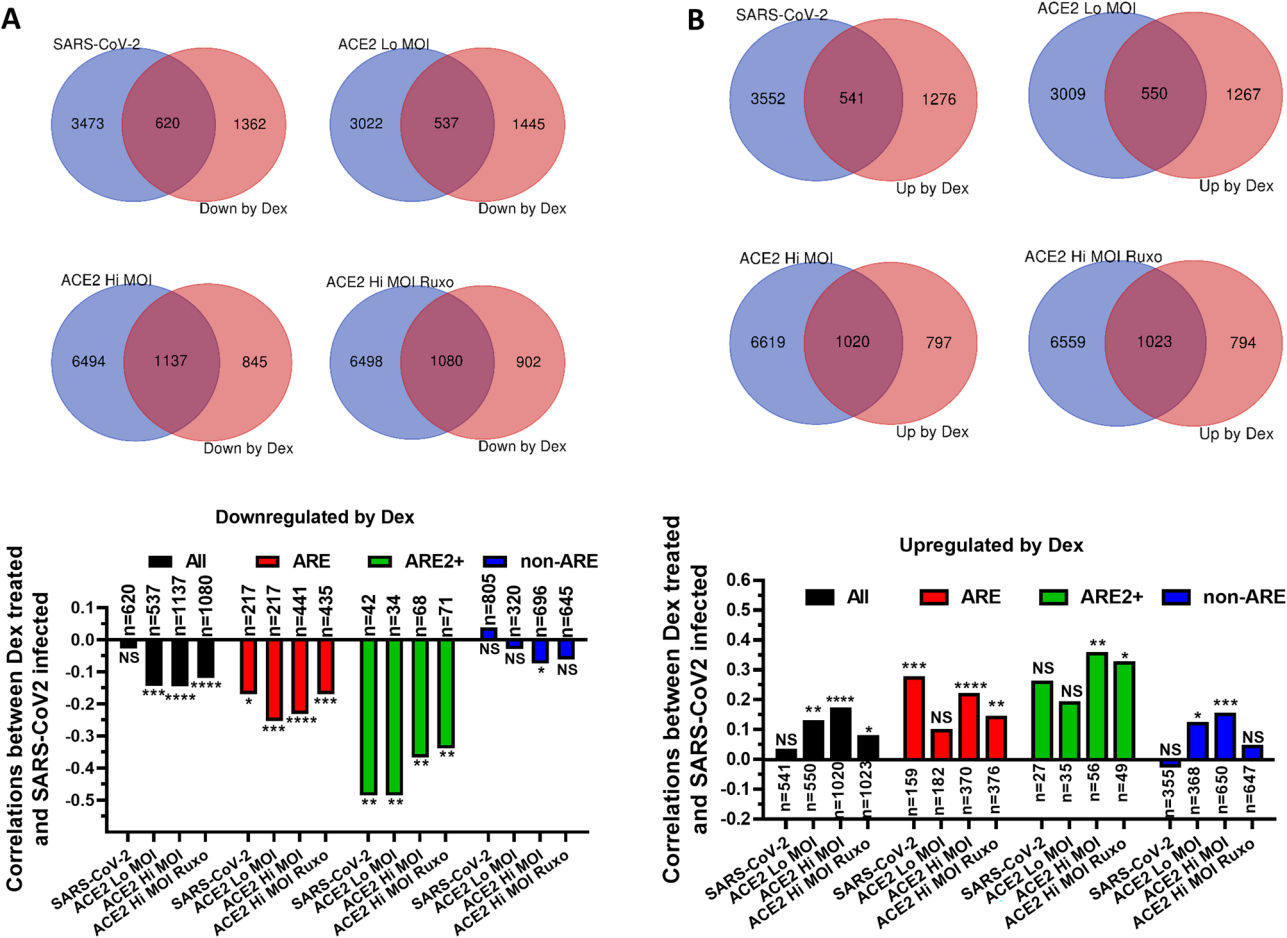
ARE-mRNAs that are downregulated by Dex tend to be upregulated by SARS-CoV-2 infection in A549 cells. (**A**) Upper panel. Venn diagrams depicting the cross between the significantly downregulated ARE mRNAs after Dex treatment in A549 cells and mRNAs that were differentially expressed in different forms of SARS-CoV-2 infected A549. As labelled; SARS-CoV-2 A549 cells were directly infected with SARS-CoV-2, “ACE2 Lo MOI” refers to A549 cells expressing the ACE2 receptor and infected with Low Multiplicity of infection. ACE2 Hi MOI refers to A549 cells expressing the ACE2 receptor and infected with High Multiplicity of infection. ACE2 “Hi MOI Ruxo” were treated with Ruxolitinib. Lower panel: the spearman correlations between mRNAs in Dex-treated and SARS-CoV-2 infected A459 cells were determined and displayed in the graph. “All” refers to all common mRNAs. “ARE” refer to mRNAs that contain AREs. “ARE2+” refer to mRNAs that are of clusters 2 + containing two or more overlapping AUUUA pentamers, “non-ARE” refers to mRNAs that do not contain AREs. (**B**) Same as A but with genes that were upregulated by Dex-treatment. The sample numbers and significance of the spearman correlations are displayed ns p > 0.05, * p < 0.05, ** p < 0.01, *** p < 0.001.

Abhay Sharma reported that a set of genes regulate in opposite directions between SARS-CoV-2 infections and Dex-treatment^32^. Based on this finding, we tested if ARE-containing genes are more likely to regulate in opposite directions compared to non-ARE mRNAs between Dex-treatment and SARS-CoV-2 infections. The observations made in Fig. 2 suggest that AREs should always lead to destabilization after Dex-treatment even when the ARE-containing mRNA is upregulated. Therefore, a destabilizing Dex effect on AREs that is independent of transcriptional noise could only be observed in the downregulated ARE-mRNAs. Consequently, mRNAs were separated between downregulated and upregulated groups after Dex-treatment. The significantly downregulated genes in Dex treated cells were selected and crossed with genes that were significantly differentially expressed in SARS-CoV-2 infected A549 cells (Padj < 0.05). The SARS-CoV-2-dependent differential expression was from Blanco-Melo et al.^33^. The authors performed four different types of infections: (i) unmodified A549 cells were infected with SARS-CoV-2, or (ii) A549 cells overexpressing the ACE2 receptor with Low Multiplicity of infection (Lo MOI) or (iii) cells expressing ACE2 with high MOI, or (iv) cells expressing ACE2 Hi MOI treated with interferon signaling inhibitor Ruxolitinib (Ruxo). Crossing the mRNAs that were downregulated by Dex and those that are differentially expressed in the different reiterations of A549 infections by SARS-CoV-2 resulted with a lists of common transcripts (Fig. 3A upper panel). The spearman correlations between the downregulated mRNAs in Dex-treated and SARS-CoV-2 infected A549 cells were determined and displayed in a graph (Fig. 3A, lower panel). A tendency to a weak negative correlation was observed when all transcripts were analyzed (Fig. 3A, lower panel, black). Next, we determined the correlations with ARE-mRNAs they turned out to be slightly more negative compared to all transcripts (Fig. 3A, lower panel, red). The strongest negative correlation was with complex ARE-mRNAs that are of clusters 2+ that have two or more overlapping AUUUA pentamers (down to − 0.49 in SARS-CoV-2 and ACE Lo MOI) (Fig. 3A, lower panel, green). The correlation of non-ARE mRNAs was either not significant or weak (Fig. 3A, lower panel, blue). Comparable and reproducible results were observed with the four different types on SARS-CoV-2 infections: SARS-CoV-2, ACE2 Lo MOI, ACE2 Hi MOI and ACE2 Hi MOI Ruxo (Fig. 3A lower panel). On the other hand, transcripts that were upregulated by Dex positively correlated with transcripts that were differentially expressed after SARS-CoV-2 infections but to a weaker extent, presumably due to transcriptional induction (Fig. 3B). The gene symbols and Log2FCs of the 2+ ARE-mRNAs that are downregulated in Dex-treated and differentially expressed in SARS-CoV-2 infected cells are available in supp. Table S3a.

### ARE-mRNAs that are downregulated by Dex in A549 cells tend to be upregulated in COVID-19 patients

**Figure 4:**
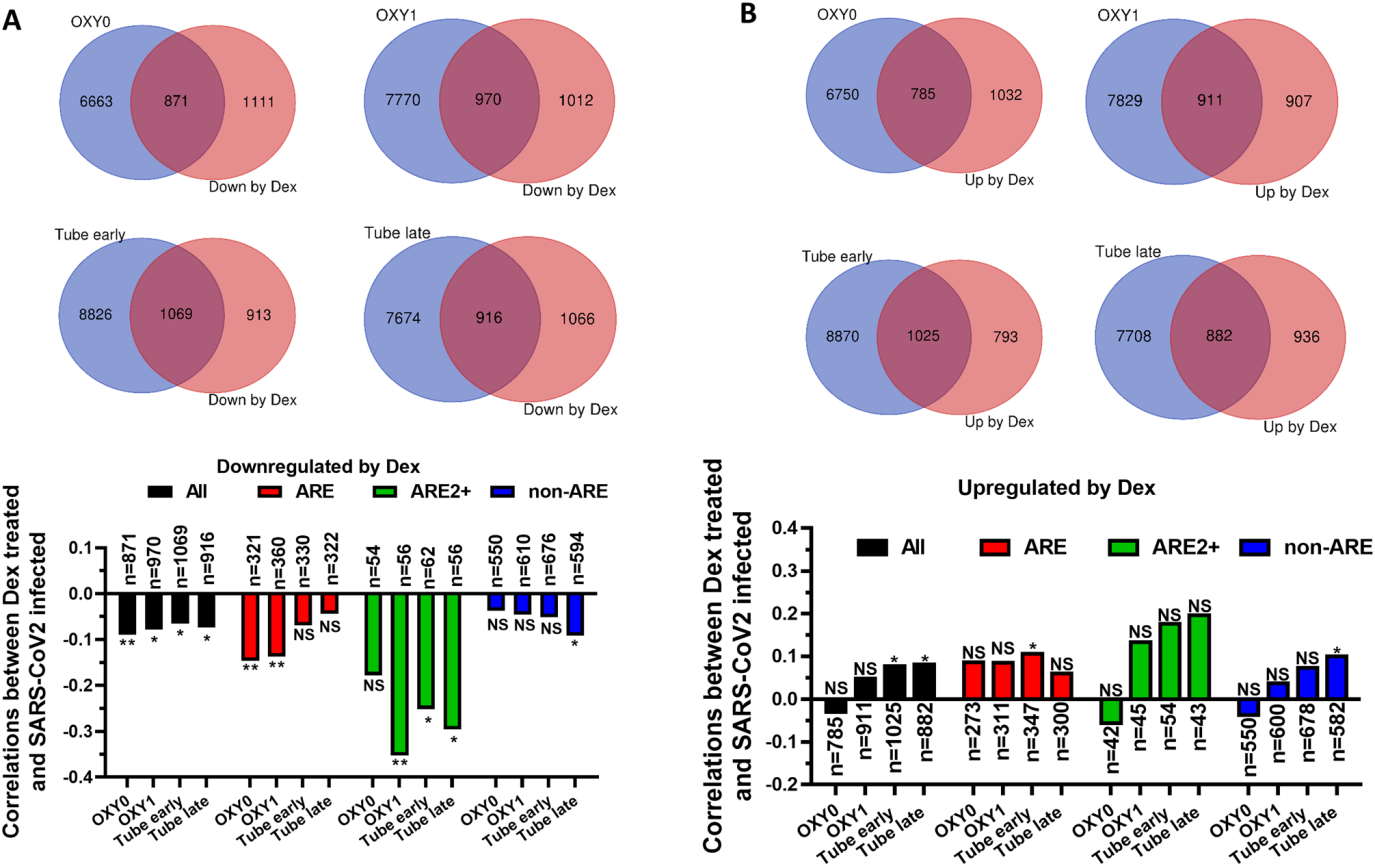
ARE-mRNAs that are downregulated by Dex in A549 tend to be upregulated in COVID-19 patients. (**A**) Upper panel. Venn diagrams depicting the cross between the significantly downregulated ARE mRNAs after Dex treatment in A549 cells and significantly differentially expressed genes in COVID-19 patients compared to healthy individuals. The patients were split into four groups according to the level of respiratory failure: no oxygen support requirements (OXY0), received oxygen but no need for mechanical ventilation (OXY1), required mechanical ventilation within the first 7 days in hospital (TUBE early) and were sampled after 7 days (TUBE late). Lower panel: the spearman correlations between the differential expression of mRNAs in Dex-treated A549 cells and COVID-19 patients were determined and displayed in the graph as indicated. The numbers of common transcripts (n) are displayed; “all” refers to all common mRNAs, “ARE” refers to common mRNAs that contain AREs, “ARE2+” refers to common mRNAs that have two or more overlapping AUUUA pentamers. Non-ARE refers to common mRNAs that do not contain AREs. (**B**) Same as A but with mRNAs that were upregulated by Dex-treatment. The significance of the correlations are displayed ns p > 0.05, *p < 0.05, ** p < 0.01, *** p < 0.001.

ARE-mRNAs are more likely to be upregulated in the blood of COVID-19 patients than the rest of the transcriptome^34,35^. In a large transcriptomic clinical study, Bibert et al. classified COVID-19 patients into four groups according to the level of respiratory failure: no oxygen support requirements (OXY0), received oxygen but no need for mechanical ventilation (OXY1), required mechanical ventilation within the first 7 days in hospital (TUBE early) and were sampled after 7 days (TUBE late). A similar analysis to the one described above was performed with patient RNA-seq data. The COVID-19-dependent differentially expressed mRNAs were crossed with the significantly downregulated genes after Dex-treatment in A549 cells (Fig. 4A upper panel). The common mRNAs had weak negative correlations between Dex-treatments and their levels in all four COVID-19 patient groups (Fig. 4A lower panel, black). ARE-mRNAs had also a tendency to negatively correlate especially in OXY0 and OXY1, the mildest forms of the disease (Fig. 4A lower panel, red). The ARE2+ mRNAs that have two or more overlapping AUUUA pentamers had a higher tendency to negatively correlate between Dex-treatment and COVID-19 infections, especially in OXY1 (ρ = − 0.35) (Fig. 4A lower panel, green). Non-ARE mRNAs abundance had weak and not significant correlations between Dex-treatments and their levels in all four COVID-19 patient groups (Fig. 4A lower panel, blue). The significantly upregulated transcripts by Dex in A549 cells, including ARE-mRNAs, had weak and not significant correlations with their differential levels in COVID-19 patients (Fig. 4A lower panel). The gene symbols and Log2FC of the downregulated 2+ ARE-mRNAs in Dex-treated and differentially expressed in COVID-19 patients is available in supp. Table S3b. Overall, the results of COVID-19 patients are weaker than in SARS-CoV-2 infected A549 cells but with similar patterns. In both case the ARE2+ group displayed the highest negative correlations between mRNAs that were reduced by Dex-treatment and SARS-CoV-2 infections. (Figs. 3 and 4).

### The effect of GC treatment on ARE-mRNAs in COVID-19 patients

**Figure 5:**
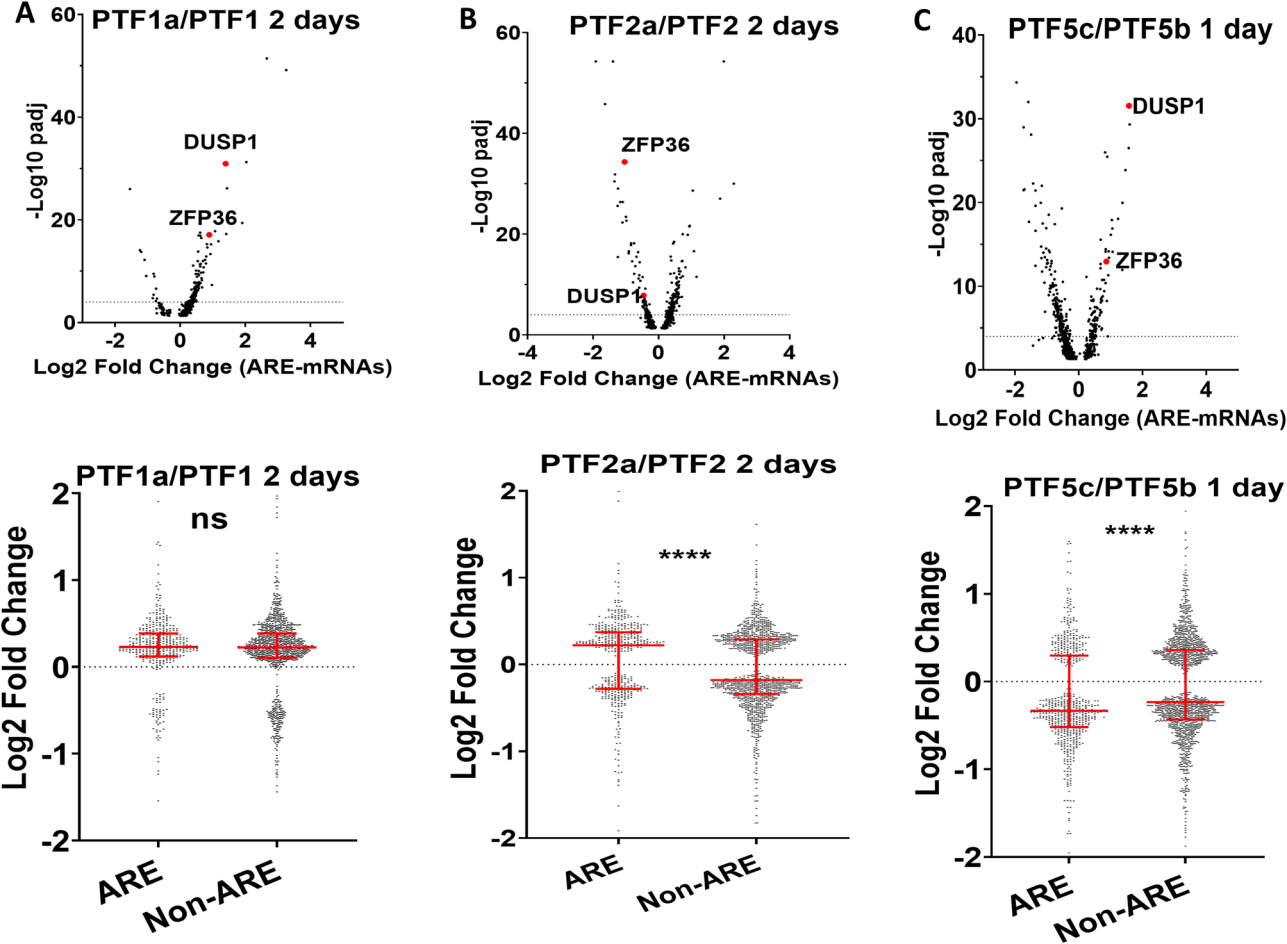
Differential expression of DUSP1, ZFP36 and ARE-mRNAs in COVID-19 patients treated with GCs. (**A**–**C**) scRNA-seq data from blood monocytes of three COVID-19 patients (PTF1, PTF2 and PTF5b) treated with daily GCs doses for different days as indicated (PTF1a, PTF2a, PTF5c). Significantly differentially expressed mRNAs between untreated and GC treated samples (padj < 0.05) were selected and split between ARE-containing (ARE) and those that do not contain AREs (Non-ARE). The significantly differentially expressed ARE–mRNAs were subjected to a volcano plot (-Log 10 of padj vs Log2 Fold Change) (upper panel), the dotted line represents log10 padj = 4. *DUSP1* and *ZFP36* were among the significantly differentially expressed mRNAs and are labelled and colored in red. (Lower panel) Dex-dependent Log2 Fold Change dot plot is displayed with median and interquartile range of ARE and non-ARE mRNAs. ns not significant P > 0.05, **** P < 0.0001.

Jeong et al. performed a single cell (sc) RNAseq study on the blood of COVID-19 patients treated with GCs (either Methylprednisolone or Dex). The study included six patients that were sampled pre and post glucocorticoid treatment, designated as PTF1, PTF2, PTF4, PTF5, PTM1 and PTM4 (GSE188172). Blood samples were labeled with patient symbol and an additional one letter suffix; for instance, PTF1 treated for 2 days with GC is labelled as PTF1a. The authors reported that the highest levels of GC-dependent differential expression was observed in monocytes compared to other immune cells^36^. Therefore, the ARE-mRNA analysis was performed in monocytes. We used the ICARUS 2.0 portal to determine the fold change of mRNAs after GC treatment in monocytes^37^. The authors reported overall high levels of heterogeneity and variations in the response to GCs among individual patients. In agreement, the differential expression of ARE-mRNAs responses investigated here was also highly heterogeneous between patients (Fig. 5A, supp. Fig. S1). Still, some interesting observations could be made, from instance, in patient PTF1, *ZFP36* and *DUSP1* were amongst the mRNAs that are most significantly upregulated after daily GC treatments for 2, 4, 8, 11, and 15 days, similar to in vitro data in A549 cells (Fig. 5A, supp. Fig. S1). The upregulation of *ZFP36* and *DUSP1* however did not correspond with a preferential down-regulation of ARE-mRNAs. In PTF2, *ZFP36* and *DUSP1* were also differentially expressed, but were surprisingly downregulated after GC treatment (Fig. 5B, supp. Fig. S1). This correlated with an expected comparative upregulation of ARE-mRNAs especially after 2 days of treatment (Fig. 5B). In PTF4, PTM1 and PTM4 the levels of *ZFP36* and *DUSP1* were weakly or not significantly differentially expressed after GC treatment corresponding with no major differential expression of ARE mRNAs (Supp. Fig. S2). For patient PTF5, scRNA seq is available 1 day after GC treatment (PTF5C/PTF5B). *ZFP36* and *DUSP1* were among the mRNAs that were most significantly upregulated. In addition, ARE-mRNAs were more downregulated compared to non-ARE mRNAs (Fig. 5C). In conclusion, the results from GC-treated patients are highly variable and heterogeneous, however, some aspects were similar to the in vitro findings like the differential expression of *ZFP36* and *DUSP1* mRNAs. This did not always correlate with the expected effects on ARE-mRNAs probably due to the complex nature of the response to the infection and treatments where other multiple factors may be involved.

## Discussion

The molecular mechanism of the destabilizing effect of GCs on cellular ARE-containing mRNAs is well investigated and understood; they induce the un-phosphorylated and active form of the ARE-binding and decay promoting protein ZFP36. This results in accelerated decay and reduced translation of several pro-inflammatory ARE-containing mRNAs. Typically, such effects were discovered on model ARE-containing mRNAs like *TNF, CXCL8* and *PTGS2*^16–18,22,28,30^.

Here, publically available RNAseq data were used to conduct a transcriptomic study on the effect of Dex-treatment on ARE-containing mRNAs in human A549 lung cancer cells. At a global scale, ARE-mRNAs had a tendency to be more downregulated compared to mRNAs that lack AREs. Still, a proportion of ARE-mRNAs were upregulated by Dex-treatment like *ZFP36, DUSP1* and *PTGER4*. mRNA stability experiments by ActD chase implied that also the upregulated mRNAs are destabilized by Dex. The chase experiments were initiated 2 h after Dex-treatment and were completed 2 h post ActD treatment, spanning a total of 4 h. During this period, the stability of the induced ARE-mRNAs was reduced. The time course experiments on the other hand, show that during the same 4-h time-period the steady state levels of *ZFP36, DUSP1* and *PTGER4* mRNAs were increasing. Therefore, the observed increase must be due to Dex-dependent increase in transcription; its impact must be stronger than destabilization and is masking its effects. On the other hand, Dex destabilizes and reduces the levels of the pro-inflammatory ARE containing mRNAs like *CXCL8, CXCL1* and *PTGS2*^9,38,39^. It appears that the same ZFP36/DUSP1-dependent mechanism that destabilizes and reduces the levels of pro-inflammatory ARE-mRNAs ends up also destabilizing induced ARE-mRNAs that express anti-inflammatory mediators. This destabilization appears counterproductive for the anti-inflammatory drugs; a possible explanation is that the GC-induced destabilization is an inevitable default process that targets all ARE-mRNAs even if not desired. Alternatively, excessive sustained induction of *ZFP36, DUSP1, PTGER4* and other ARE-mRNAs by Dex may be detrimental. Therefore, it is tightly regulated at the post-transcriptional level to counter transcription, resulting in mild induction. As a result, Dex ends up downregulating many pro-inflammatory ARE-mRNAs and upregulating several that reduce inflammation. Inflammatory stimuli like IL1 on the other hand, induce both the pro-inflammatory and anti-inflammatory ARE-mRNA.

Tiedje et al. used the iCLIP technique to identify ZFP36/TTP targets in bone marrow-derived mouse macrophages at a transcriptomic scale. The authors compared the affinity to AREs of wild type ZFP36 with the ZFP36-AA mutant. This mutant has two serine to alanine substitutions at two MK2 phosphorylation sites and therefore cannot be phosphorylated/inhibited by MK2. Twenty top mRNAs that are targets of ZFP36 were identified, 16 of which contained AREs. Interestingly, ten of the 16 were also modified by Dex-treatment according to the observations here namely *CCL2, CDKN1A, CXCL2, DUSP1, GDF15, IER3, MDM2, NFKBIA, TNFAIP3* and *TNFSF9*. Remarkably, those targets were more likely to bind ZFP36-AA compared to WT-ZFP36. This is in agreement with the observations of this study since ZFP36 that is induced by Dex is presumably not phosphorylated and should act like ZFP36-AA^11^.

The observations discussed above imply that an “isolated” post-transcriptional effect of Dex on ARE-mRNAs should be detectable only in the downregulated group, since upregulation is likely the result of transcription that masks destabilization. Therefore, mRNAs that were downregulated by Dex were investigated separately from mRNAs than that were upregulated by Dex. Indeed, opposite directions of regulation between Dex and SARS-CoV-2 infections were only significant in ARE-mRNAs that were downregulated by Dex. The highest negative correlations were observed with complex clusters 2+ AREs that have two or more overlapping AUUUA repeats are more likely to respond to stimuli.

At a global scale, the ARE-mRNA upregulation response appeared more pronounced in COVID-19 patients with mild disease (OXY0)^35^. However, the negative correlation between ARE2+ mRNAs that were downregulated by Dex and induced by COVID-19 was strongest in patients with more severe disease. These observations suggest that Dex could be reducing the levels of key mRNAs at the post-transcriptional level that are especially upregulated in patients with severe COVID-19.

The effect of GC treatment on the ARE-transcriptome of the COVID-19 patients was variable and heterogeneous. In three out of six patients, *ZPF36* and *DUSP1* were amongst the ARE-mRNAs that were most significantly induced after GC. However, only in one patient this upregulation correlated with a preferential downregulation of ARE-mRNAs compared to non-ARE. In contrast with the in vitro results, *DUSP1* and *ZFP36* mRNAs were downregulated in one patient after GC treatment, this correlated with an expected comparative upregulation of ARE-mRNAs. These observations highlight variable, patient-specific responses to GC treatments and a likely complex dynamic balance between the effects of infection and GC treatments.

This analysis of available transcriptomic RNA-seq data provides a global view and improved understanding of the effect of GCs on ARE-mRNAs levels in human cells. GCs are likely to have a universal destabilizing effect on ARE-mRNAs, but a heterogeneous overall outcome on their abundance due to variations in response to infections and the combined effects of transcription and post-transcription.

## Methods

### RNA-seq data sources on glucocorticoid response

The lung cancer cell line A549 has been extensively used for the investigation of glucocorticoid response in human cells. An NCBI search with the terms “A549 and glucocorticoid” retrieves 425 publications, 67 of which had GEO depositions. Further search refinements for “RNA-seq, A549 and glucocorticoid” in Gene Expression Omnibus (GEO) resulted in 15 records. Two datasets contained relevant direct analysis of the effect of GCs on global mRNA levels: GSE104714 and GSE159546^23,24^ (Supplemental Flowchart 1). The GSE104714 dataset contained densely sampled mapped read counts of A549 cells treated with Dex. Specifically, A549 cells were treated with Dex at 3, 5, 7, 9, and 11 h in two replicates for each time point along with time-specific ethanol vehicle controls^23^. The DESeq2 version 1.38.3 in R, was used to identify differentially expressed mRNAs. All time point replicates and corresponding controls were combined in a single paired analysis comprising a total of ten Dex-treatments and ten ethanol treatment controls (DESeq2 results, design matrix and code available in supplemental Table S1). Genes with Padj < 0.05 Benjamini and Hochberg adjustment for multiple testing, from DESeq2 results, were considered differentially expressed with statistical significance^40,41^. Dataset GSE159546 contained mapped read counts of two control and treatment replicates incubated for 8 h with hydrocortisone. This dataset was processed similarly in DESeq2 (supplemental Table S2)^24^. The ARED-plus AU-rich element database was used to select ARE genes^2^.

### Correlations between glucocorticoid treatments and COVID-19 infections

The differential expression of mRNAs (Log2FC values) were correlated between Dex treatment and COVID-19 infections by spearman correlation. RNA-seq data from A549 cells infected with SARS-CoV-2 virus were from GSE147507. We previously used DESeq2 to determine differentially expressed genes^33^. RNA-seq data from the blood of COVID-19 patients with different severity levels were from Bibert et al. The authors deposited the mapped counts in (data.mendeley.com/datasets/8wxhhykfnh/2)^34^. We previously used the deposited data from this study to compute the Log2 Fold Changes and significance^35^. The Dex-response data from GSE104714 was available with transcript identity; the ENSEMBL portal was used to assign gene names for each transcript (Supplemental Table 1). In some cases, several transcripts were assigned to a single gene. The different transcripts of the same genes had very similar Dex-repsonse patterns. Therefore, we selected the differentially expressed transcript with the highest statistical significance (Lowest padj) as a representative of the gene. Lists of common genes with significant Log2FC values between the two conditions (Dex treatment and SARS-CoV-2 infection) were prepared and spearman correlations with its significance were determined in graph prism.

### COVID-19 patients treated with GCs

Single cell RNA-seq data of COVID-19 patients treated with GCs were from (GSE188172)^36^. The mapped mRNA counts from patients’ sampled pre and post GC treatment (PTF1, PTF2, PTF4, PTF5, PTM1 and PTM4) were downloaded from NCBI GEO database GSE188172. The ICARUS 2.0 portal was used to compute differential expression in blood monocytes^37^. Default functions for data integration and corrections were applied. Monocytes were labeled using Single R and DICE immune cell expression data. The ARED-plus AU-rich element database was used to select ARE genes^2^.

### Statistical analysis and display

The GraphPad Prism software was used for data display and statistical analysis. Non-parametric Man-Whitney test was used to assess the significance of differentially expressed ARE and non-ARE mRNAs. Differences in ARE-mRNA proportions were tested by two sided fisher’s exact test in graph prism. Spearman correlations of transcript levels after Dex treatments or SARS-CoV-2 infections were determined in Graph Prism. Western blot bands were quantified in Image Lab (BioRad). Venn diagrams were performed in (https://bioinformatics.psb.ugent.be/webtools/Venn/).

### Cells, treatments, protein lysates and western blotting

A549 cells were purchased from American Type Culture Collection (ATCC) and cultured in DMEM (Invitrogen), supplemented with 10% FBS and antibiotics (Invitrogen). Dexamethasone sodium phosphate 4 mg/ml was from Simplist/USA. Dilutions were made in cell culture medium to reach a final concentration of 100 nM treatments. For western blots, cells were directly lysed in 1 × SDS sample buffer (Invitrogen). Lysates were sonicated to shear DNA, and equivalent lysate levels were loaded onto SDS PAGE gel, blotted to nitrocellulose membrane, and probed with antibodies to ZFP36, and GAPDH. Affinity-purified ZFP36 rabbit polyclonal antibody was custom-made with Genescript and was used previously^5^. GAPDH, Phspho-p38 antibody was purchased from Cell Signaling. P38 antibody was purchased from Santa Cruz. IL1 alpha was from R&D systems, and was used to treat the cells at a 3ng/ml concentration.

### RNA preparation, real-time PCR, and actinomycin-D chase

Total RNA was extracted with TRI reagent (Sigma). Reverse transcription was performed using Superscript II and Oligo dT primer (Invitrogen). Real-time PCR TaqMan primer sets including the FAM-labeled *ZFP36, PTGS2, CXCL8, CXCL1, DUSP1, PTGER4, TFCP2L1, DNAJC15, IER3, ENC1* as Target genes and VIC-labeled GAPDH as internal control were purchased from Applied Biosystems. Real-time PCR was performed using the CFX96 cycler (BioRad). For mRNA half-life determination, the cells were treated with 100 nM Dexamethasone for 2 h. After 2 h of treatment, Actinomycin-D (10µg/ml) was added to shut off transcription at different time point, and then total RNA was extracted with TRI reagent and qRT-PCR was performed as above. Decay curves were plotted using GraphPad Prism software, mRNA half-life was estimated by one phase exponential decay.

### Supplementary Information

Supplementary Table S1.

Supplementary Table S2.

Supplementary Table S3.

Supplementary Information 1.

Supplementary Information 2.

Supplementary Figure S1.

Supplementary Figure S2.

## Data Availability

The datasets used and/or analysed during the current study are available in this published article and its supplementary information files and from the corresponding author on reasonable request.
