# Supplementary material for: Global analysis of the abundance of AU-rich mRNAs in response to glucocorticoid treatment
Source: Sci Rep. 2024 Jan 9;14:913. doi: 10.1038/s41598-024-51301-6 (PMC10776588; doi:10.1038/s41598-024-51301-6)
Supplement: Supplementary file 4 — Supplementary Information 1. [file 41598_2024_51301_MOESM4_ESM.pdf]

Supplemental File F1

Uncropped Figures 2D

D

Dex (h) 0 1 2 4 6 8

ZFP36

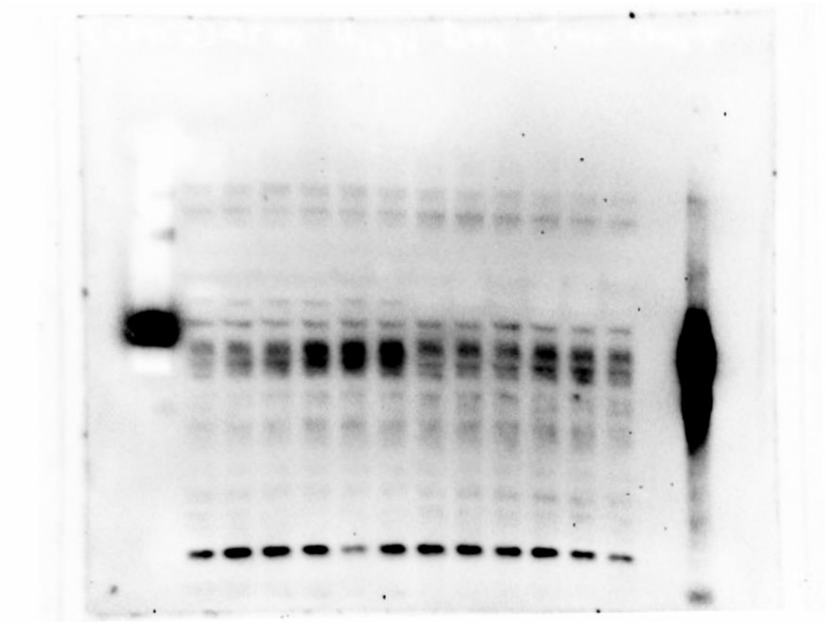

GAPDH

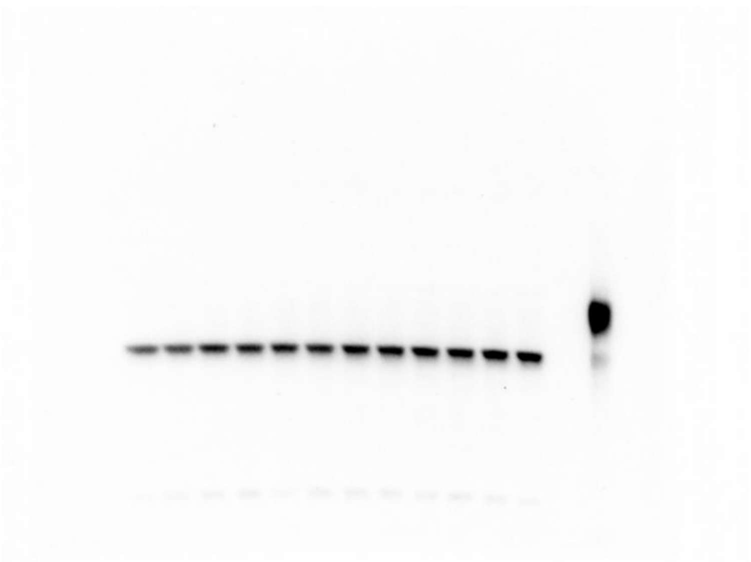

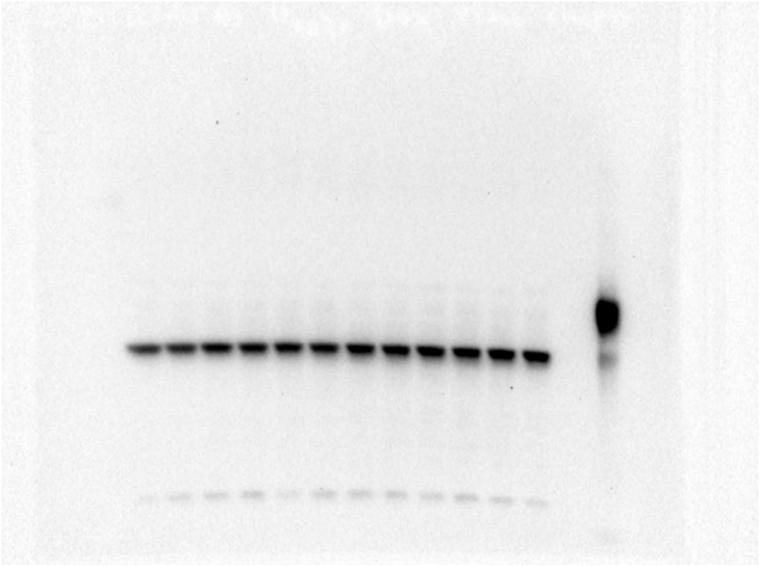

GAPDH No contrast

A549 cells were treated with either 100nM of Dex or 3ng/ml IL1 for the indicated time points and western blot analysis was performed to assess ZFP36, phosphorylated p38 (P-p38), p38 and GAPDH levels

Supplemental File F1

Uncropped Figures 2E

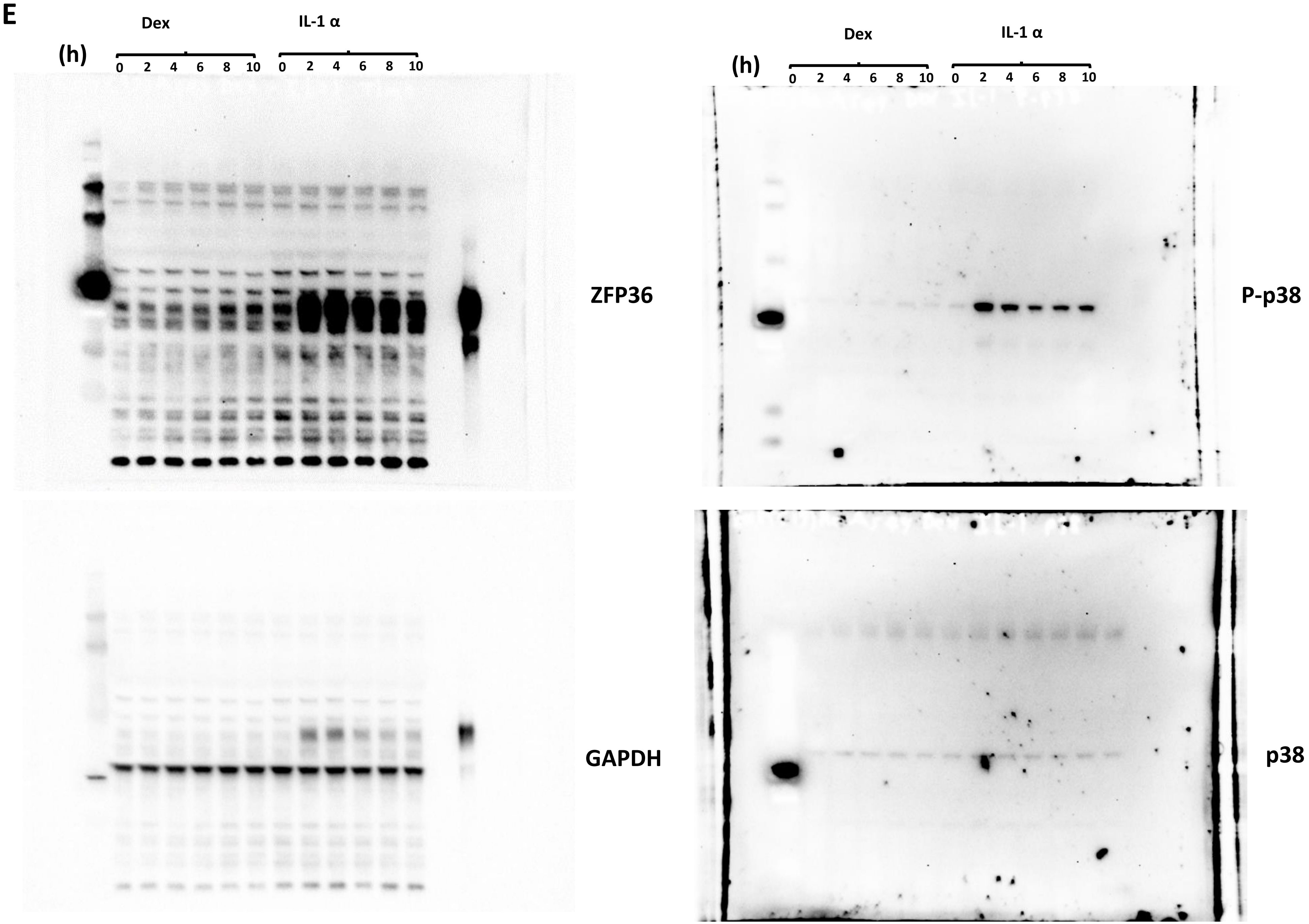

A549 cells were treated with either 100nM of Dex or 3ng/ml IL1 for the indicated time points and western blot analysis was performed to assess ZFP36, phosphorylated p38 (P-p38), p38 and GAPDH levels
