# Supplementary material for: Global analysis of the abundance of AU-rich mRNAs in response to glucocorticoid treatment
Source: Sci Rep. 2024 Jan 9;14:913. doi: 10.1038/s41598-024-51301-6 (PMC10776588; doi:10.1038/s41598-024-51301-6)
Supplement: Supplementary file 5 — Supplementary Information 2. [file 41598_2024_51301_MOESM5_ESM.pdf]

Flow diagram for the selection of RNA-seq datasets from A549 cells treated with Glucocorticoids

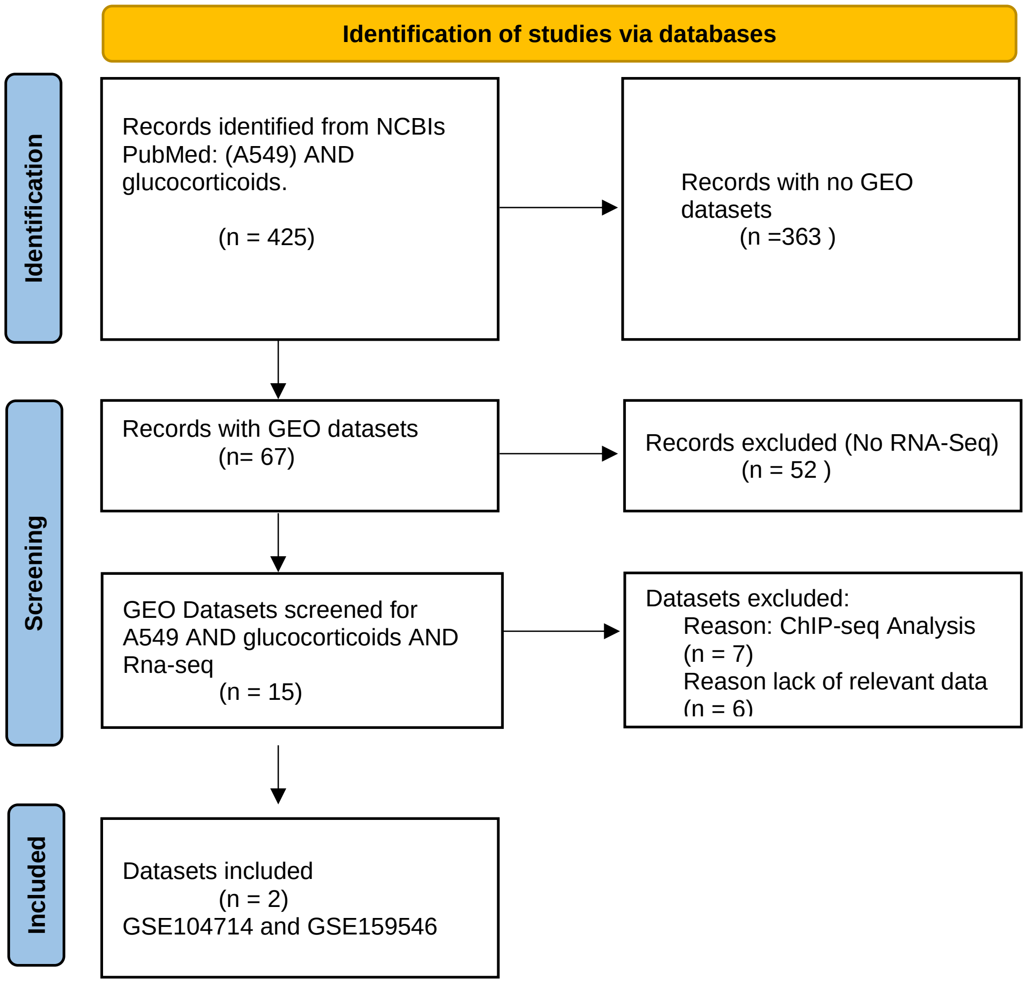

Inspired by Page et. al. with modifications.

Page MJ, McKenzie JE, Bossuyt PM, Boutron I, Hoffmann TC, Mulrow CD, et al. The PRISMA 2020 statement: an updated guideline for reporting systematic reviews. BMJ 2021;372:n71. doi: 10.1136/bmj.n71

http://www.prisma-statement.org/
