# Supplementary material for: Global analysis of the abundance of AU-rich mRNAs in response to glucocorticoid treatment
Source: Sci Rep. 2024 Jan 9;14:913. doi: 10.1038/s41598-024-51301-6 (PMC10776588; doi:10.1038/s41598-024-51301-6)
Supplement: Supplementary file 6 — Supplementary Figure S1. [file 41598_2024_51301_MOESM6_ESM.pdf]

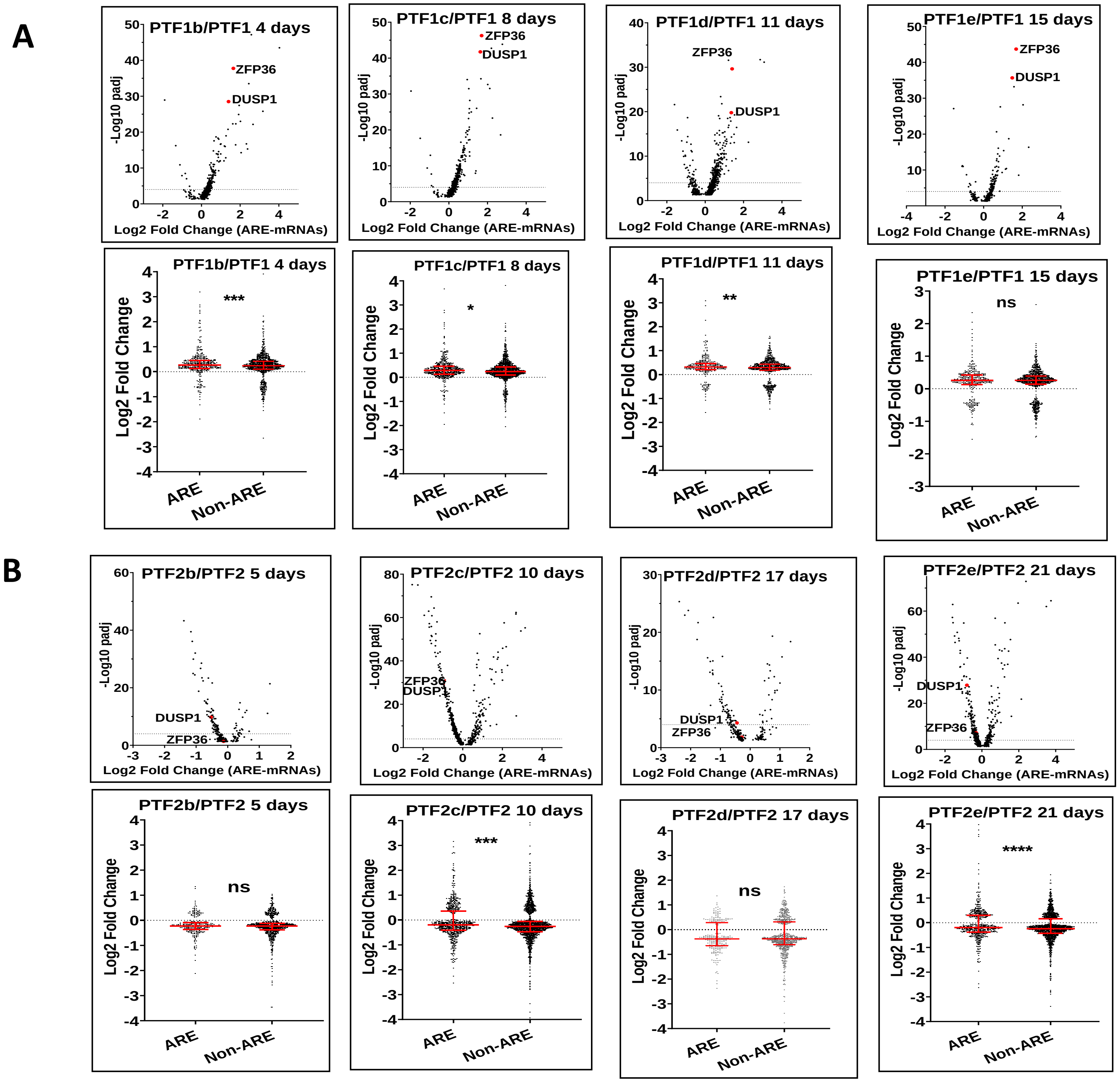


**Differential expression of DUSP1, ZFP36 and ARE-mRNAs in COVID-19 patients treated with GCs. (A-B)** scRNA seq data from two COVID-19 patients (PTF1 and PTF2) treated with daily GCs doses for different days as indicated. Significantly differentially expressed mRNAs between untreated and GC treated samples (padj<0.05) were selected and split between ARE containing (ARE) and those that don't contain AREs (Non-ARE). The significantly differentially expressed ARE–RNAs were subjected to a volcano plot (upper panel), (-Log 10 of padj vs Log2 fold change), the dotted line represents –log10 padj=4. DUSP1 and ZFP36 were among the most significantly differentially expressed mRNAs and are labelled and colored in red. Dex dependent Log2 Fold Change dot plot is displayed with median and interquartile range of ARE and non-ARE mRNAs (lower panel). ns not significant $P > 0.05$, * $P < 0.05$, ** $P < 0.01$, *** $P < 0.001$, **** $P < 0.0001$.
