# Supplementary material for: Global analysis of the abundance of AU-rich mRNAs in response to glucocorticoid treatment
Source: Sci Rep. 2024 Jan 9;14:913. doi: 10.1038/s41598-024-51301-6 (PMC10776588; doi:10.1038/s41598-024-51301-6)
Supplement: Supplementary file 7 — Supplementary Figure S2. [file 41598_2024_51301_MOESM7_ESM.pdf]

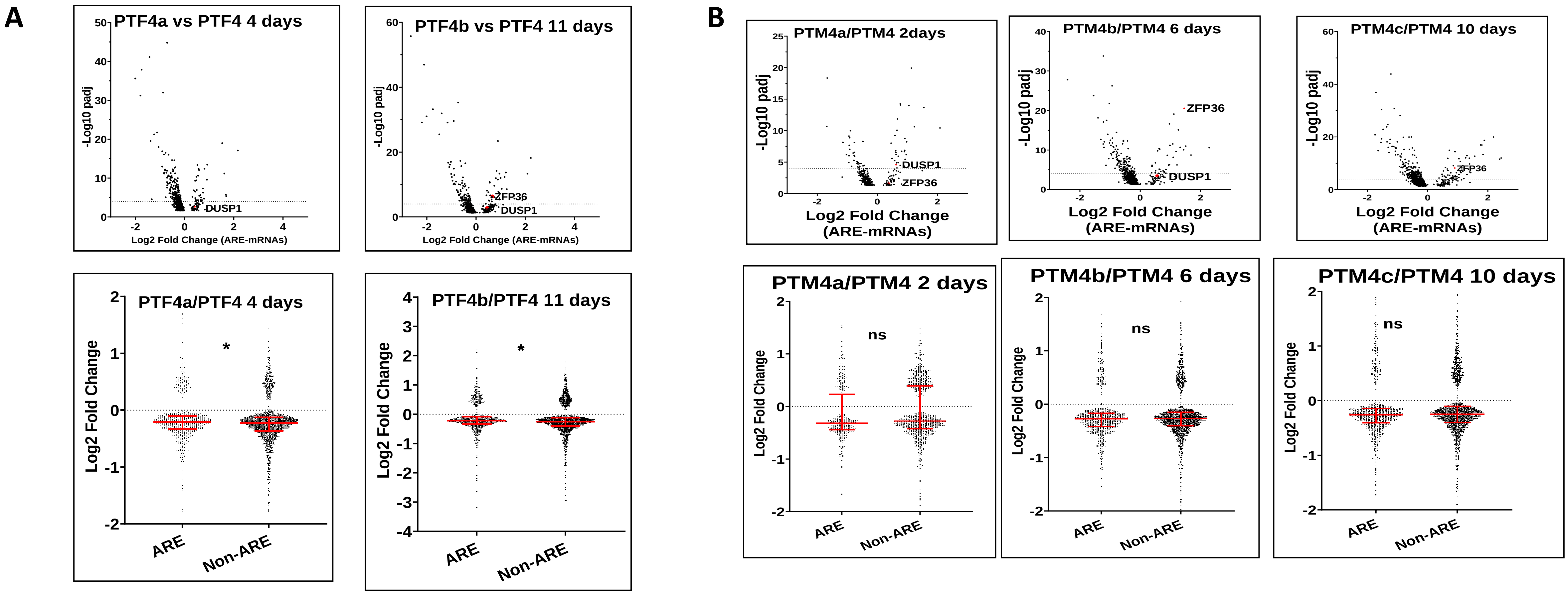

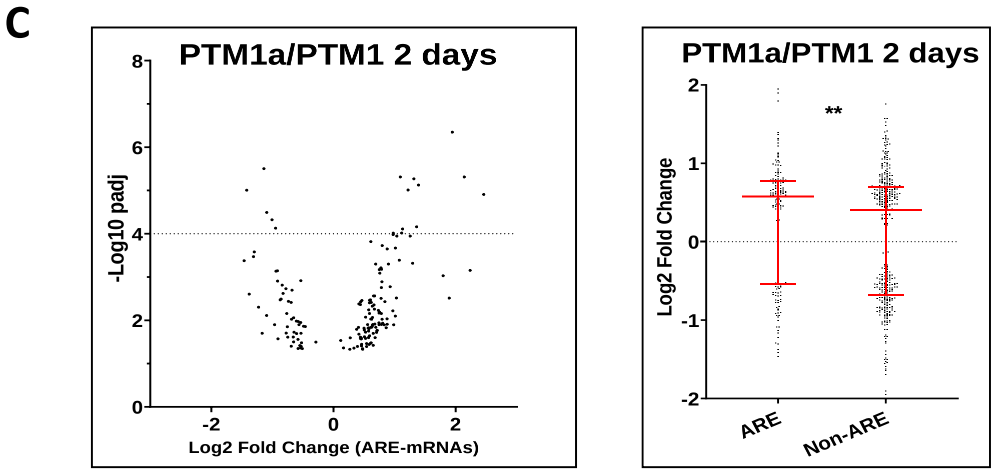

Differential expression of DUSP1, ZFP36 and ARE-mRNAs in COVID-19 patients treated wit GCs. (A-C) scRNA seq data from three COVID-19 patients (PTF4, PTM4 and PTM1) treated with daily GCs doses for different days as indicated. Significantly differentially expressed mRNAs between untreated and GC treated samples (padj<0.05) were selected and split between ARE containing (ARE) and those that don't contain AREs (Non-ARE). The significantly differentially expressed ARE–RNAs were subjected to a volcano plot (upper panel), (-Log 10 of padj vs Log2 fold change), the dotted line represents –log10 padj=4. In case DUSP1 and ZFP36 were among the significantly differentially expressed mRNAs, they are labelled and colored in red. (lower panel) Dex dependent Log2 Fold Change dot plot is displayed with median and interquartile range of ARE and non-ARE mRNAs. ns not significant P > 0.05, * P < 0.05, ** P < 0.01, *** P < 0.001, **** P < 0.0001.
